# Understanding Daptomycin Resistance Mechanisms and Treatment Challenges in *Enterococcus faecium* Infection: A Case Series

**DOI:** 10.3390/antibiotics15030243

**Published:** 2026-02-26

**Authors:** Sangeeta Nair-Collins, Gabriel Godart, Nipakumari Patel, Vidit Yadav, Kelly Larimore, Jordan D. LeGout, Rohit Chitale, Ravi Durvasula, Justin Oring

**Affiliations:** 1 Division of Infectious Diseases, Department of Medicine, Mayo Clinic, Jacksonville, FL 32224, USA; nair-collins.sangeeta@mayo.edu (S.N.-C.); larimore.kelly@mayo.edu (K.L.); chitale.rohit@mayo.edu (R.C.); durvasula.ravi@mayo.edu (R.D.); oring.justin@mayo.edu (J.O.); 2 Department of Radiology, Mayo Clinic, Jacksonville, FL 32224, USA

**Keywords:** daptomycin-resistant *Enterococcus faecium*, multidrug-resistant *Enterococcus faecium*, *Enterococcus faecium*, post-liver transplant, liver disease, DRE*fm*

## Abstract

Daptomycin-resistant *Enterococcus faecium* (DRE) poses an increasing therapeutic challenge, particularly in immunocompromised patients and solid organ transplant recipients. Surveillance data from the Centers for Disease Control and Prevention indicate that approximately 6.5% of *E. faecium* isolates are daptomycin-resistant, underscoring the need for heightened clinical vigilance, particularly for prompt identification and treatment. In this case series, three patients with advanced liver disease, including two status post orthotopic liver transplantation, are described who developed DRE during treatment for bloodstream infection. These cases illustrate the dynamic nature of antimicrobial susceptibility under daptomycin exposure and highlight the contributions of persistent source control issues, intravascular infection, and altered host factors to treatment failure. All patients were successfully managed by escalating to combination therapy with high-dose daptomycin and ceftaroline, alongside appropriate source control. This series emphasizes the importance of periodic susceptibility reassessment during daptomycin therapy and cautions clinicians against assuming sustained susceptibility in patients with prolonged bacteremia or complex infections. Early recognition of evolving resistance and timely therapeutic adjustment may improve outcomes in this high-risk population.

## 1. Introduction

Enterococci are a group of Gram-positive, facultatively anaerobic cocci that are commonly found in the gastrointestinal systems of both humans and non-human animals, as well as in soil, water, and plants, and can cause a range of illnesses, including urinary tract infections, bacteremia, endocarditis, cellulitis, and intra-abdominal infections. Enterococci were initially classified as part of Streptococcus Group D, but in the 1980s, they were reclassified as a new genus, Enterococcus, and renamed as *Enterococcus faecalis* and *Enterococcus faecium* [1]. These two organisms are the predominant causative agents of human enterococcal disease [2].

### 1.1. The History of Antimicrobial Resistance

Enterococci are intrinsically resistant to many antibiotics, including cephalosporins, penicillin, and clindamycin. A study of antibiotic-naïve patients confirmed intrinsic resistance to penicillin and cephalosporins [3]. In 1989, researchers at the Medical College of Pennsylvania reported that about one-third of *E. faecium* isolates were penicillin-resistant [4]. Ampicillin resistance is primarily due to the presence of penicillin-binding protein 5 (pbp5), which raises the minimum inhibitory concentration (MIC). Higher levels of pbp5 were associated with reduced antibiotic susceptibility [5].

*E. faecium* isolates commonly show high resistance to ampicillin, approximately 85% [6], making vancomycin the first-line treatment. Vancomycin-resistant enterococci (VRE) were initially detected in the UK and France in the mid-1980s and quickly spread across Europe and the United States. Within seven years, the incidence rose from 0.3% to 7.9%. Five gene clusters—VanA, VanB, VanC, VanD, and VanE—are responsible for vancomycin resistance, but only VanA, VanB, and VanD are found in *E. faecium*. VanA, the most prevalent in the U.S. and Europe, confers high-level inducible resistance even at low vancomycin levels [7]. The global rise in VRE continues to pose serious clinical challenges, with resistance rates ranging from 8% to 34% [8]. At our institution, only 48% of *E. faecium* isolates remain susceptible to vancomycin.

### 1.2. The Emergence of Daptomycin Resistance

Linezolid is the only FDA-approved medication for VRE infections. Although daptomycin lacks this approval, it is frequently used due to its bactericidal activity, which disrupts bacterial membrane potential and halts DNA, RNA, and protein synthesis. While resistance remains rare (0.6%), it poses significant treatment challenges due to limited treatment options and high mortality in bloodstream infections [9]. One institutional study reported daptomycin resistance rates of up to 15% [10].

The Clinical and Laboratory Standards Institute (CLSI) has updated the daptomycin MIC to 2–4 for dose-dependent susceptibility (SDD) against *E. faecium* [11]. In this paper, we show that this updated MIC may still be associated with treatment failure. The first reported case of resistance during treatment occurred in 2005, when a patient’s MIC increased from 2 to >32 while receiving daptomycin for bacteremia [12]. Though not fully understood, resistance has been linked to increased cell wall thickness, higher surface charge, reduced depolarization after exposure, and electrostatic repulsion of the daptomycin–Ca^2+^ complex from the bacterial membrane [13,14].

Here, we describe three cases of treatment-induced daptomycin resistance in *E. faecium* in patients with liver pathology.

## 2. Cases Presentations

### 2.1. Case #1

A 60-year-old male with a past medical history most remarkable for Crohn’s disease diagnosed 25 years ago, and end-stage liver disease secondary to cholangiocarcinoma, was admitted to our facility in July 2023 to undergo an orthotopic full-size liver transplant (OLT) with a Roux-en-Y anastomosis. Notably, he completed a course of Rifaximin 500 mg every 12 h as prophylaxis against hepatic encephalopathy.

The preoperative evaluation, including abdominal magnetic resonance imaging (MRI) and magnetic resonance cholangiopancreatography (MRCP), identified a distended gallbladder with wall ulceration, concerning for perforation, which was managed with a percutaneous transhepatic cholecystostomy tube. Cultures from the drainage grew *Citrobacter freundii* complex, for which the patient was treated with double-strength Trimethoprim-Sulfamethoxazole for 14 days every 12 h.

Microbiological analysis of the explanted liver grew Vancomycin-Resistant *Enterococcus faecium* (VRE) with daptomycin susceptibility (MIC = 0.5 mcg/mL) (Table 1). Additionally, the culture revealed two fluconazole-resistant Candida tropicalis colonies, indicating a polymicrobial infection.

To manage the VRE infection, the patient was started on IV daptomycin (6 mg/kg) for 7 days. Simultaneously, IV caspofungin 50 mg q24h was continued for Candida, which had been initiated due to the prolonged surgical procedure. By the end of July 2023, approximately two weeks post-transplant, he was clinically stable and discharged.

Three days after discharge, the patient developed neutrophilic leukocytosis and, the following day, returned to the emergency department with an acute onset of severe symptoms, including rigors, nausea, vomiting, diarrhea, and a syncopal episode. On examination, he was hemodynamically unstable, exhibiting tachycardia (131 beats per minute), tachypnea (25 breaths per minute), and hypotension (87/56 mm Hg). He also had a fever, peaking at 38.1 °C.

Initial laboratory investigations revealed severe lactic acidosis (lactate 6.7 mmol/L) and marked leukocytosis (white blood cell count 41.9 × 10^9^/L). This was accompanied by a pronounced left shift, indicated by an absolute neutrophil count (ANC) of 40.46 × 10^9^/L. The patient was admitted to the ICU for suspected sepsis and close monitoring.

Blood cultures drawn from two peripheral sites revealed a polymicrobial infection in one bottle, with growth of VRE, Candida glabrata, and MDR Citrobacter freundii.

**Figure 1:**
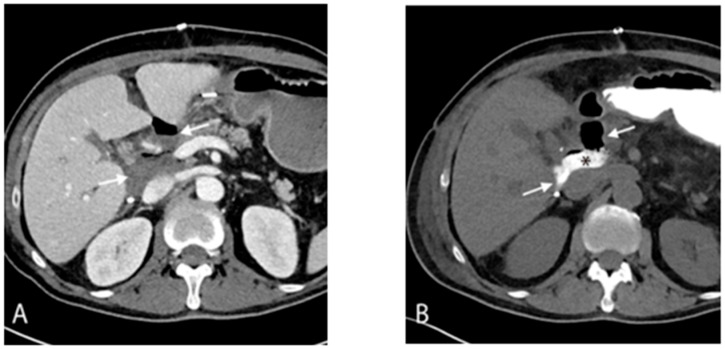
Contrast-enhanced CT (**A**) and CT with oral contrast (**B**) show an air-containing fluid collection (white arrows) centered in the hilum of the transplant liver, contacting the inferior vena cava. There is extravasation of oral contrast into the fluid collection (*), confirming viscus perforation of most likely duodenal source. This imaging finding suggests a persistent gastrointestinal source with proximity to major vascular structures, providing a mechanistic explanation for ongoing polymicrobial bacteremia despite antimicrobial therapy.

The patient’s antimicrobial regimen was intensified to treat the polymicrobial infection. Daptomycin was increased to 12 mg/kg IV, and caspofungin was escalated to 100 mg IV every 24 h. Additionally, IV meropenem (1 g q8h) and IV gentamicin (1 mg/kg q8h) were added to augment treatment of the high-grade VRE bacteremia. CT imaging of the abdomen and pelvis was performed to evaluate the source of infection, revealing a subhepatic fluid collection (Figure 1) likely secondary to a viscus perforation originating from the duodenum.

Interventional radiology (IR) drained this fluid. The aspirate cultures grew VRE, MDR Citrobacter Freundii, and Candida Glabrata. Linezolid 600 mg IV q12h and voriconazole 200 mg PO q12h were added due to persistent high-grade bacteremia and candidemia. Additionally, a transthoracic echocardiogram and transesophageal echocardiogram were obtained to rule out infective endocarditis.

**Figure 2:**
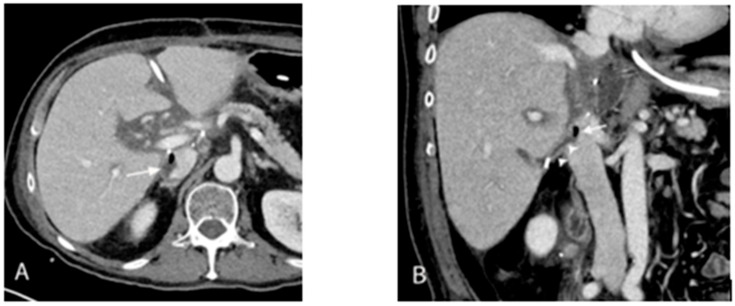
Axial (**A**) and coronal (**B**) contrast-enhanced CT images show an air-containing thrombus in the IVC (arrows), compatible with septic thrombophlebitis. A causative enterocaval fistula tract extending from the abscess of duodenal origin is demonstrated (arrowheads). These findings identify a vascular nidus of infection, supporting the need for combined source control and prolonged antimicrobial therapy and contextualizing the persistence of bacteremia.

A month after the initial admission (mid-August 2023), the persistence of high-grade VRE bacteremia despite resolution of candidemia necessitated further evaluation. A repeat CT of the abdomen and pelvis revealed the development of septic thrombophlebitis of the inferior vena cava (IVC) due to an enterocaval fistula from the site of duodenal perforation and abscess as the source of the polymicrobial bacteremia (Figure 2). The patient underwent endoscopic closure with surgical clipping to close the duodenal defect.

At the same time, repeated daptomycin susceptibility testing revealed that the isolate had developed daptomycin resistance. The treatment regimen was revised in consultation with clinical pharmacists, adding ceftaroline 600 mg IV every 8 h for synergy. This adjustment in antibiotics, along with appropriate source control via surgical closure, led to negative blood cultures after 4 days. Because endoscopic closure and antimicrobial escalation occurred in temporal proximity, causality could not be attributed to either intervention alone.

After resolution of the bacteremia, linezolid and gentamicin were discontinued. The patient’s treatment was continued with a combination regimen of IV daptomycin (12 mg/kg q24h) and IV ceftaroline (600 mg q8h) for a total of 21 days, ending in mid-September 2023, during which the patient remained hospitalized. Subsequent surveillance blood cultures obtained while the patient remained hospitalized became persistently negative and did not later demonstrate daptomycin-resistant *E. faecium*, indicating a favorable clinical response.

In this case, persistent daptomycin-resistant *Enterococcus faecium* bacteremia was associated with a duodenal perforation complicated by an enterocaval fistula and septic thrombophlebitis. Blood cultures cleared after combined source control and escalation to combination antimicrobial therapy with daptomycin and ceftaroline. The patient completed an extended course of therapy without recurrence of daptomycin-resistant *E. faecium* on subsequent surveillance cultures. At follow-up, the patient remained clinically stable, with no evidence of recurrent bacteremia. A timeline of the patient’s clinical course is summarized in Table 2.

### 2.2. Case #2

A 68-year-old female with a history of end-stage liver disease secondary to autoimmune hepatitis, complicated by hepatocellular carcinoma (HCC), status post Y-90 and orthotopic full-size deceased donor liver transplant (OLT) 3 years ago. The patient is CMV donor-positive/recipient-negative, EBV recipient-positive, and indeterminate on QuantiFERON gold.

One month post-transplant, she was hospitalized for choledocholithiasis within the recipient common bile duct, which was managed with endoscopic retrograde cholangiopancreatography (ERCP) and complete biliary sphincterotomy. However, her condition deteriorated and was complicated by high-grade MRSA bacteremia with MRSA culture-positive loculated ascites. She underwent interventional radiology (IR) guided drainage of the collection for source control. She was started on vancomycin 1250 mg IV every 12 h inpatient and subsequently discharged on a four-week course of daptomycin 6 mg/kg IV daily, initial MIC = 1.

Three months later (approximately four months post-transplant), she presented with fever and generalized body aches and was found to have high-grade ampicillin-susceptible *E. faecium* bacteremia (MIC ≤ 2). She was started on ampicillin 2 g IV q4h. The daptomycin MIC was 4, although our microbiology laboratory did not initially perform the test.

Given persistent high-grade bacteremia, she underwent a transesophageal echocardiogram, which was unrevealing. A magnetic resonance cholangiopancreatography (MRCP) showed interval development of a thrombus within the 8th hepatic segment and a portal vein branch, with sites of hepatic infarction, raising concern for an endovascular source of infection to explain her persistent high-grade bacteremia. Her antimicrobial regimen was broadened to ampicillin 4 g IV every 4 h and ceftriaxone 2 g IV every 12 h. However, despite appropriate therapy, *E. faecium* bacteremia persisted. Repeat susceptibility testing demonstrated a change in the antimicrobial susceptibility profile of *Enterococcus faecium*, with subsequent isolates exhibiting resistance to both ampicillin (MIC > 8) and vancomycin (MIC > 16) (Table 3).

As a result, ampicillin was discontinued, and she was started on daptomycin 8 mg/kg IV daily and ceftaroline 600 mg IV every 12 h. The latter was added to mitigate the risk of daptomycin resistance. Gentamicin 80 mg IV every 24 h was also added for synergy until she became culture negative. She was continued on triple therapy until blood cultures stayed negative for 72 h. At that time, she was de-escalated to dual therapy with daptomycin 10 mg/kg daily and a two-week course of gentamicin 3 mg/kg IV daily.

Her three-week follow-up labs showed elevated liver enzymes (AST 97 U/L, ALT 149 U/L, and ALP 571 U/L), prompting an MRI/MRCP. The imaging revealed a hepatic biloma measuring 4.7 × 2.5 × 2.3 cm. The IR aspirated fluid culture grew *E. faecium*, with restored susceptibility to daptomycin, now with MIC ≤ 0.5. After completing dual therapy with gentamicin and daptomycin, she was de-escalated to monotherapy with daptomycin 10 mg/kg daily.

Approximately a month later (six months post-transplant), she developed abdominal distension. A repeat MRCP showed an enlarging biloma now measuring 5.1 × 3.2 cm. An IR-guided percutaneous drain was placed into the biloma, and cultures yielded *E. faecium* colonies that reverted to daptomycin resistance (MIC = 8).

As a result, daptomycin 10 mg/kg daily therapy was extended for an additional 2 weeks, and ceftaroline 600 mg IV every 8 h was added (total therapy duration of 10 weeks). A follow-up MRCP showed a reduction in biloma size.

Post completion of therapy two weeks later, the patient’s clinical condition deteriorated, and she returned to the ED with fever (T_max_ of 101.9 °F), acute abdominal pain, and foul-smelling biliary drainage. A CT scan of the abdomen and pelvis with IV contrast showed a new 1.5 cm hypodensity in segment V of the liver, suggesting a hepatic abscess.

She was initially started on high-dose daptomycin 12 mg/kg/hour, but eventually switched to eravacycline 100 mg IV every 12 h for four weeks to cover other gut organisms. After completion of therapy with eravacycline, she was transitioned to lifelong suppressive antibiotic therapy with amoxicillin 500 mg PO TID and doxycycline 100 mg PO BID. An ERCP performed one week later showed findings suggestive of ischemic cholangiopathy with no findings of an abscess. At 1 year post-treatment follow-up, she has not experienced any recurrence of bloodstream infections or fluid collections.

**Figure 3:**
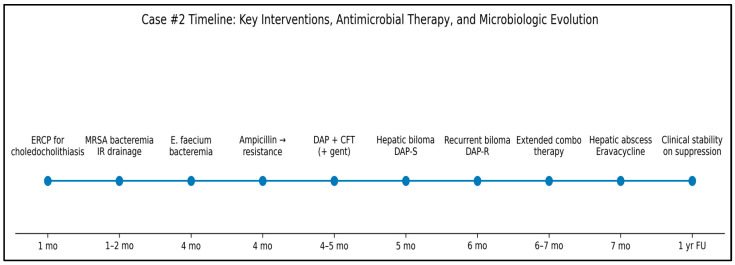
Timeline of key clinical events, antimicrobial therapy, and microbiologic evolution in Case #2. The figure highlights recurrent Enterococcus faecium infections with dynamic changes in daptomycin susceptibility in the setting of biliary complications and repeated source control interventions following liver transplantation.

Given her complex medical history—including MRSA bacteremia, persistent DRE infections, ischemic cholangiopathy, chronic immunosuppression, and the challenges with available antibiotic options—suppressive antibiotic therapy would serve as a long-term strategy to minimize bacterial growth and reduce the burden of infection. The key clinical events are summarized chronologically in Figure 3.

### 2.3. Case #3

A 54-year-old female with a medical history remarkable for systemic lupus erythematosus on rituximab, common variable immunodeficiency (CVID) complicated by idiopathioc thrombocytopenic purpura (ITP) status post splenectomy in 1998, roux-en-Y gastric bypass, recurrent *Clostridium difficile* infections managed by a total abdominal colectomy and end ileostomy in 2022 and protein malnutrition requiring open placement of gastrostomy admitted for planned gastric bypass reversal and ileostomy takedown.

The postoperative course was complicated by wound dehiscence requiring exploratory laparotomy and fascial dehiscence repair, with an intraoperative note showing extensive intra-abdominal purulence. She was subsequently started on piperacillin/tazobactam. Intraoperative cultures grew vancomycin-resistant *Enterococcus faecium* (MIC >16 mcg/mL), *Pseudomonas aeruginosa*, and *Enterobacter cloacae*. *E. faecium* was noted to be susceptible to daptomycin, with an MIC of 2 µg/mL (Table 4). Based on the susceptibility profile, she was started on daptomycin 6 mg/kg daily and meropenem 1 g IV q 8 h for management of vancomycin-resistant *Enterococcus faecium* and polymicrobial peritonitis. Upon discharge, she was continued on a six-week course of daptomycin, IV levofloxacin 750 mg daily, and IV ceftolozane-tazobactam 1.5 g IV q8h.

Her postoperative course was complicated by bacteremia with MSSA, Klebsiella pneumoniae, and Staphylococcus lugdunensis, all requiring IV antibiotic treatment appropriately managed by Infectious Disease. Given recurrent bacteremia, an intra-abdominal source of infection was suspected. She underwent a whole-body PET-CT, which was negative for any noticeable nidus of intra-abdominal infection.

Approximately a year after these recurrent episodes of bacteremia, she was admitted for completion of proctectomy, revision of ileostomy, and exploratory laparotomy. No intraoperative cultures were taken. Post surgery, she developed worsening leukocytosis and abdominal pain. A CT abdomen and pelvis with IV contrast showed a partially loculated fluid collection with subsequent placement of an IR-guided drain. Fluid culture was positive for *E. faecium*, multidrug-resistant *Escherichia coli*, and *Metamycoplasma hominis*. *E. faecium* was now noted to be resistant to daptomycin, with an MIC of 8 mcg/mL.

She was discharged on a planned four-week course of linezolid 600 mg PO twice daily and ertapenem 1 g every day. A repeat CT of the abdomen and pelvis showed findings concerning a persistent fluid collection. However, the biopsy was not consistent with a fluid collection and instead showed myofibroblastic proliferation, most consistent with scar formation. Follow-up imaging showed reaccumulation of a rim-enhancing presacral fluid collection measuring approximately 4.0 × 3.4 cm, despite prior IR-guided drainage. Cultures were positive for *Metamycoplasma hominis*, for which she was started on a four-week course of doxycycline 100 mg twice daily.

## 3. Discussion

Though daptomycin resistance in *Enterococcus* spp. is low, less than 1%, the emergence of daptomycin-resistant *Enterococcus* spp. poses significant challenges to medical providers and necessitates the development of newer antibiotics. The first reported case of daptomycin resistance during vancomycin-resistant *Enterococcus faecalis* treatment was reported in 2005, and its incidence has gradually increased [15].

We present three cases of patients initially susceptible to daptomycin (MIC = 2) who developed resistance during treatment. The emergence of resistance in these cases could be attributed to several factors, including acquired mutations, immunosuppression, medication, altered daptomycin pharmacokinetics, sequestered foci, and the potential for biofilm formation.

### 3.1. Mechanism of the Development of Daptomycin Resistance

The mechanisms of daptomycin resistance in *E. faecium* are not fully understood. Still, mutations in the YycFGHIJ stress-sensing system, LiaFSR pathway, and enzymes involved in phospholipid biosynthesis, such as cardiolipin synthetase (cls) and GDPD, are commonly implicated. These mutations alter the bacterial stress response, increasing membrane thickness and modifying phospholipid composition, thereby hindering daptomycin’s membrane interaction, both of which contribute to reduced efficacy [16,17,18].

Further research is needed to fully understand how these mutations translate to clinical resistance and how they may be overcome. For example, in one of our patients, we demonstrated a transient increase in susceptibility to daptomycin. De novo cases of daptomycin resistance have been reported in isolated cases among patients with no prior exposure to daptomycin, though the mechanism remains unclear [19].

### 3.2. Impact of Metabolic and Immunosuppressive Factors on Daptomycin Resistance

#### 3.2.1. Role of Immunosuppression in POST-OLT Subgroup

Post-solid organ transplant infection risk is influenced by socioeconomic factors and the patient’s net state of immunosuppression, including post-transplant immunosuppressive regimens and baseline clinical factors such as hyperglycemia, renal dysfunction, and indwelling device use [20]. In the liver transplant patient population, *E. faecium* is a key pathogen causing intra-abdominal infections, including cholangitis, peritonitis, bloodstream infections, and urinary tract infections [21]. In this subgroup, the risk of developing DRE is further increased by pre-transplant VRE colonization, prior antibiotic use, prolonged hospital stays, interventional procedures, biliary complications, and the potential need for surgical re-exploration [22].

The development of daptomycin resistance is influenced by prolonged drug exposure, high bacterial loads, altered pharmacokinetics (PK), and potential dosing challenges in patients with renal replacement therapy and in those with poor source control [23]. A lack of proper source control has been observed among patients who developed daptomycin resistance. A study found that 19% of patients developed daptomycin resistance after initial daptomycin therapy, and many had poor source control of their infections. Poor source control likely leads to prolonged antibiotic therapy, thereby increasing the likelihood of resistance [21].

While solid organ transplant recipients receive similar immunosuppressive regimens, they are known to experience a high incidence of bacterial infections, particularly within the first month post-transplant [24]. Notably, Liver transplant recipients account for a significant proportion of DRE infections, indicating an increased vulnerability to these resistant pathogens in this population [25].

#### 3.2.2. Pharmacokinetics of Daptomycin

Daptomycin is highly protein-bound (approximately 90%; binding is not concentration-dependent), primarily excreted through the kidneys and, to a lesser extent, via bile [26]. Decreased creatinine clearance, abnormal bile secretion, fluid shifts, and decreased albumin levels may increase the volume of distribution (V_d_) and the unbound fraction of daptomycin [27]. Unbound daptomycin redistributes throughout the extracellular space and vascular tissue, resulting in lower concentrations in target regions [28].

Post-OLT ICU stay is associated with complications such as vascular or biliary complications, persistent cholestasis, and the use of an immunosuppressive regimen [29]. These factors may increase the V_d_, thereby reducing drug concentrations at the targeted site. Additionally, studies have shown that severe infections with Gram-positive cocci can alter daptomycin PK, specifically by increasing V_d_, resulting in suboptimal drug levels at the infection site [30]. According to Bender et al., the emergence of daptomycin resistance may be associated with suboptimal drug levels at the site of infection, thereby increasing the risk of selecting for resistant mutants [31].

### 3.3. Role of Sequestered Foci and Biofilm Formation

Biofilm formation is another key factor contributing to VRE persistence [32]. Tissue-related biofilm infections are more prevalent in immunocompromised patients and those with underlying chronic conditions, such as cardiovascular disease, diabetes, cancer, or skin barrier damage, particularly when the infection is severe or occurs early in the disease course [33]. In such cases, these sequestered infection sites serve as protected environments that enable pathogens like *E. faecium* to evade immune responses and resist antibiotic treatment through various mechanisms (described below), thereby promoting the emergence of resistance. In our case series, fluid collections in the abdominal space (case#1 and case#3) and a biliary tract biloma (case#2) served as reservoirs for the VRE strain, allowing continued bacterial propagation despite appropriate antibiotic therapy and source control measures. Both patients ultimately developed daptomycin resistance, suggesting that biofilm formation within these sequestered foci contributed to treatment failure.

The mechanism of biofilm-associated antimicrobial resistance lies in the biofilm matrix, which impedes antibiotic diffusion and retains bacterial load through slower growth rates and phenotypic variation within biofilms, further reducing antibiotic efficacy. Additionally, Biofilm-forming bacteria can secrete enzymes, such as proteases, that facilitate immune evasion and contribute to the development of biofilms that inactivate antibiotics [33]. Biofilm-associated pathogens exhibit a resistant phenotype, which complicates eradication efforts and contributes to bacterial evasion of antibiotics and phagocytosis [34].

In *E. faecium*, biofilm formation is strongly associated with increased antimicrobial resistance. 75% of *E. faecium* isolates were noted to be biofilm producers [35]. Adaptations promoting intestinal colonization in *E. faecium* contribute to its resistance. For example, exposure to bile acids in the mammalian GI tract triggers a morphotype switch from diplococci to long chains. This switch enhances biofilm formation via the autolysin AtIA, which is associated with increased bacterial aggregation and colonization, particularly in VRE-colonized mice. In these mice, genetic analysis has identified mutations in the LiaFSR and YycFG stress-response systems in clinical *E. faecium* isolates, which are associated with the development of daptomycin resistance [36]. Jun-Hong Ch’ng et al. suggest that biofilms formed by *enterococcus* species act as a reservoir for antibiotic resistance genes, which facilitate horizontal transmission of resistant genes both intra- and inter-species. Antibiotic resistance genes identified include Ebp, Epa, pCF10, and PrgABC, which regulate biofilm formation. These factors promote bacterial aggregation, gene transfer, and daptomycin sequestration, thereby complicating therapeutic interventions and facilitating the sustained survival of resistant *E. faecium* strains [37]. Fallah et al. found no correlation between the esp, ebpR, and asa1 genes and biofilm formation, despite detecting these genes in 84.2%, 91.2%, and 100% of enterococcal isolates, respectively [35]. In response to daptomycin, the EpaOX and Epal genes mediate polysaccharide production and maturation in *E. faecalis*, which sequesters daptomycin from its target. However, these mechanisms of biofilm-associated resistance in *E. faecium* have not been extensively studied and require further investigation [38].

In vitro pharmacokinetic and pharmacodynamic studies have demonstrated that daptomycin doses of at least 8 mg/kg/day are required for effective bactericidal action, with 10 mg/kg/day potentially needed to prevent the development of resistance in high-bacterial-load infections [39]. High-dose daptomycin (>8 mg/kg/day) raises concerns for rhabdomyolysis, acute renal failure, and uncertain efficacy. However, one study found that higher doses of daptomycin (≥9 mg/kg) were associated with reduced mortality in patients with VRE bacteremia [40]. Combining daptomycin with beta-lactams such as ampicillin, ceftriaxone, cefepime, ertapenem, or ceftaroline can significantly enhance daptomycin’s effectiveness. Among these, ceftaroline has been shown to remarkably reduce the MIC of daptomycin by improving its binding to bacterial membranes and enhancing its bactericidal properties [41].

A retrospective multicenter cohort study analyzed 139 cases of enterococcal bacteremia following liver transplantation (LT). 78% of cases were caused by VRE and 22% (31 cases) by DRE. They found that prior daptomycin exposure was an independent predictor of DRE bacteremia (aOR, 30.62; 95% CI, 10.087–92.955; *p* < 0.001), with 81% of liver transplant recipients with DRE bacteremia having prior post-transplant exposure to daptomycin (unadjusted OR, 27.98; 95% CI, 9.76–80.2; *p* < 0.001). Furthermore, 22/31 (71%) of patients with DRE bacteremia had a prior post-transplant VRE infection, compared with only 2/76 (2.6%) of patients with VRE bacteremia [42].

Just as we used combination therapy with daptomycin and beta-lactam, Lee et al. also observed that patients with DRE bacteremia were more likely to receive linezolid (67%), combination therapy (daptomycin/beta-lactams, daptomycin/linezolid, or triple therapies, 17%), high-dose daptomycin (defined as ≥8 mg/kg, 7%), or other therapies such as quinupristin/dalfopristin or tigecycline (10%) [41].

### 3.4. Role of Rifaximin in Daptomycin Resistance

Rifaximin, a bacterial RNA polymerase inhibitor with direct activity in the gut, is used prophylactically to prevent hepatic encephalopathy in patients with liver cirrhosis who are at higher risk for developing VREfm. The emergence of vancomycin-resistant enterococci is associated with prior antibiotic use, manipulation of the gastrointestinal tract, and the presence of indwelling catheters. Patients with liver pathology are at risk for developing recurrent intra-abdominal infections, such as spontaneous bacterial peritonitis, leading to multiple rounds of antimicrobial use. Furthermore, these patients are often treated with rifaximin.

Exposure of VREfm strains to prolonged rifaximin is associated with the development of an RpoB substitution that confers cross-resistance to daptomycin [43]. RpoB, a component of the bacterial genome, encodes the beta subunit of RNA polymerase and plays a key role in bacterial transcription. It is the site of development of multipl4069e mutations. Some, such as loss-of-function mutations, can significantly affect the function of bacterial RNA polymerase. Other mutations alter the binding site, leading to resistance. Mutations within the RpoB site have been shown to upregulate the pdr operon, leading to cell membrane remodeling and increased positive cell surface charge, thereby preventing daptomycin binding.

Using animal models, Turner et al. showed that 90% animals inoculated with VREfm and exposed to rifaximin for 7 days developed daptomycin resistance. Similar findings were noted with rifampicin. Whether these daptomycin-resistant strains persisted after discontinuation of rifamycin was not examined and may warrant future research [43].

### 3.5. Novel Therapies of DRE Management and Prospects

There is currently a paucity of data available to guide the treatment of DRE. The oxazolidinones (linezolid/tedizolid) are the primary treatment of choice against DRE. However, toxicity, including peripheral neuropathy and serotonin syndrome, and bacteriostatic activity have precluded the use of this class, particularly in cases requiring prolonged treatment.

Eravacycline is a fluorocycline and is structurally similar to tigecycline but is not subjected to the mechanisms that are responsible for tetracycline resistance, such as efflux pumps and ribosomal protection proteins. It has been shown to have susceptibility to vancomycin-resistant *E. faecium* isolates. In one study, 460 *E. faecium* blood isolates were tested, and 235 (51%) were vancomycin-resistant. 89.7% of these resistant organisms were susceptible to eravacycline [44,45]. However, whether similar efficacy is observed in DRE remains unclear and requires further study.

The novel cephalosporin, ceftobiprole, is active against ampicillin-susceptible isolates of *E. faecalis* and *E. faecium*, while only remaining active against vancomycin-resistant *E. faecalis*, but not vancomycin-resistant *E. faecium* [46].

Quinpristine-dalfopristine, a combination of streptogramin antibiotics, has shown promise in the treatment of MDR enterococcal infections. Its bactericidal activity has demonstrated efficacy against multidrug-resistant *Staphylococcus aureus* and vancomycin-resistant *Enterococcus faecium*. While not commonly used due to side effects and limited indications, quinpristine-dalfopristine can provide synergy in combination regimens and may offer a helpful option in cases of DRE when other therapies fail. While these emerging agents may provide alternative treatment options, their efficacy in treating DRE remains under evaluation [47].

Oritavancin, a lipoglycopeptide, has demonstrated in vitro activity against VREfm [48]. The activity against daptomycin-resistant enterococci was further studied by Belley et al. They evaluated two daptomycin-nonsusceptible VREfm mutant strains and exposed them to a single 3 h infusion of oritavancin. It resulted in a significant reduction in bacterial viability over 24 h, with the bacterial population rebounding after 72 h, suggesting that multiple doses of oritavancin may be necessary to adequately eradicate resistant strains.

Cross-resistance between oritavancin and daptomycin was also observed, with daily daptomycin administration leading to the emergence of mutants with cross-resistance to oritavancin, resulting in a fourfold increase in oritavancin MICs. However, when oritavancin was administered more than 24 h after daptomycin exposure, the development of cross-resistance was less likely, as most mutants were eliminated during the interval. These findings suggest that coordinating the timely administration of oritavancin after daptomycin exposure could serve as a potential salvage strategy, allowing a switch from daptomycin to oritavancin in the event of rising daptomycin MICs [48]. It is important to note that these observations are based on ongoing research, and further studies are needed to fully understand the clinical implications.

Combination therapy with daptomycin and ceftaroline has been shown to increase daptomycin binding even in non-susceptible strains. Smith et al. evaluated whether similar synergistic findings were noted when daptomycin was combined with other β-lactam antibiotics. They quantified daptomycin binding by fluorescence to determine its extent in *E. faecium*. Synergistic effects were not established with imipenem or ceftriaxone. However, other studies have shown that *E. faecium* mutations within the *LiaFSR* system restored the daptomycin MIC when combined with ampicillin [49].

### 3.6. Daptomycin vs. Tigecycline for Vancomycin-Resistant Enterococcus

Tigecycline is an FDA-approved drug for complicated intra-abdominal infections. Both daptomycin and tigecycline demonstrate excellent in vitro activity against VRE; however, daptomycin exhibits rapid bactericidal activity, whereas tigecycline is bacteriostatic [50,51]. In transplant cases with bacteremia like ours, this bactericidal activity becomes much more vital due to patients being on immunosuppressive therapy. Moreover, tigecycline comes with a ‘black-box’ warning due to increased all-cause mortality with use for FDA-approved, as well as non-approved indications compared with other antibiotics [52]. Therefore, due to its bactericidal activity, safer profile, and stronger clinical evidence, daptomycin is preferred over tigecycline for vancomycin-resistant Enterococcus infections, particularly for bacteremia and endocarditis.

### 3.7. Limitation

We acknowledge that, in the absence of whole-genome sequencing, we cannot definitively distinguish true clonal evolution of resistance from alternative explanations, such as heteroresistance or mixed infection with genetically distinct *E. faecium* strains. Nevertheless, the temporal association between antimicrobial exposure and reproducible changes in susceptibility profiles across serial clinical isolates supports the clinical relevance of these observations.

## 4. Conclusions

Daptomycin-resistant *Enterococcus faecium* (DRE) represents an emerging and clinically significant challenge, particularly among liver transplant recipients, in whom immunosuppression, altered pharmacokinetics, persistent sources of infection, and biofilm formation contribute to treatment failure and adverse outcomes. Surveillance data from the Centers for Disease Control and Prevention indicate a growing prevalence of DRE, underscoring the need for heightened clinical awareness in high-risk populations [53].

This case series highlights the dynamic nature of antimicrobial susceptibility during daptomycin therapy and reinforces the importance of early recognition and prompt reassessment when clinical response is suboptimal. Across three cases, evolving resistance patterns necessitated therapeutic escalation and, in some instances, combination antimicrobial strategies to achieve microbiologic clearance.

In the absence of established guidelines for the management of DRE infections, we recommend periodic antimicrobial susceptibility testing following initiation of daptomycin, particularly in patients with persistent bacteremia, inadequate source control, worsening clinical trajectories, or prior exposure to rifaximin prophylaxis. When linezolid is contraindicated or prolonged therapy is required, consideration may be given to combination therapy with high-dose daptomycin (10–12 mg/kg IV once daily) and ceftaroline 600 mg IV every 8 h, as illustrated in this series.

Continued investigation into resistance mechanisms, host–pathogen interactions, and strategies to disrupt biofilm formation is required to optimize treatment approaches. Equally important are robust infection prevention and antimicrobial stewardship efforts, including active surveillance, isolation precautions, and judicious antibiotic use, to limit the emergence and transmission of DRE and improve outcomes in vulnerable patient populations.

## Figures and Tables

**Table 1:** MICs are reported in µg/mL, with categorical interpretations based on CLSI breakpoints applicable at the time of testing, using broth microdilution. “Not tested” indicates antimicrobial susceptibility testing was not performed for that agent on the specified isolate. OLT = orthotopic liver transplantation.

Antimicrobial Agent	Pre-Transplant: Explanted Liver, MICs	Pre-Transplant: Blood Culture, MICs	1 Month Post-OLT: Blood Culture, MICs
Ampicillin	≤2 µg/mL (Susceptible)	Not tested	>8 µg/mL (Resistant)
Daptomycin	≤0.5 µg/mL (Susceptible)	Not tested	8 µg/mL (Resistant)
Linezolid	≤2 µg/mL (Susceptible)	≤2 µg/mL (Susceptible)	>4 µg/mL (Resistant)
Vancomycin	>16 µg/mL (Resistant)	>16 µg/mL (Resistant)	>16 µg/mL (Resistant)
Penicillin	Not tested	>8 µg/mL (Resistant)	Not tested
Gentamicin (High-level synergy)	Not tested	≤500 µg/mL (Susceptible)	Not tested

**Table 2:** Chronological summary of clinical events, microbiology, and antimicrobial therapy in Case #1.

Approx. Date (Hospital Day)	Event/Intervention	Microbiology/Cultures
July 2023 (admission) (Day 1)	Orthotopic liver transplant (OLT); explanted liver sent for culture	Explant culture: VRE isolated; daptomycin MIC = 0.5 µg/mL.
Late July (Day 9)	Initial daptomycin therapy (6 mg/kg IV daily) → clinical stabilization → discharged	—
Early August (3 days after discharge) (Day 13)	Return with neutrophilic leukocytosis → ED → sepsis next day; blood cultures drawn	Blood cultures: polymicrobial growth (VRE, *C. glabrata*, MDR *C. freundii*).
Mid-August (Day 30)	Antimicrobial escalation (daptomycin dose increased, meropenem, gentamicin, caspofungin); IR drainage of subhepatic collection	IR aspirate culture: same organisms (VRE, *C. glabrata*, MDR *C. freundii*).
Mid-August (Day 33)	CT: septic thrombophlebitis/enterocaval fistula from duodenal perforation → endoscopic closure with surgical clip	Bloodstream isolates showed daptomycin resistance on susceptibility testing.
Mid- to late-August (Day 36)	Ceftaroline 600 mg IV q8h added (in consultation); continued high-dose daptomycin	After addition of ceftaroline and endoscopic closure, surveillance blood cultures became persistently negative.
Mid-September (Day 57)	Completed extended 21-day combination course (daptomycin + ceftaroline); remained hospitalized during this period.	Sustained absence of daptomycin-resistant *E. faecium* on surveillance cultures.
Mid-October (Day 96)	Patient discharged by the Transplant & Primary care team	Sustained absence of DRE on surveillance cultures.

**Table 3:** MICs are reported in µg/mL with categorical interpretations based on CLSI breakpoints applicable at the time of testing broth microdilution. “Not tested” indicates antimicrobial susceptibility testing was not performed for that agent on the specified isolate. OLT = orthotopic liver transplantation.

Antimicrobial Agent	4 m Post-OLT: Blood Culture	4.1 m Post-OLT: Blood Culture	5 m Post-OLT: Aspirated Abdominal Fluid	6.5 Months Post-OLT Aspirated Liver Abscess Fluid	
				Isolate 1	Isolate 2
Ampicillin	≤2 µg/mL (Susceptible)	>8 µg/mL (Resistant)	>8 µg/mL (Resistant)	>8 µg/mL (Resistant)	>8 µg/mL (Resistant)
Daptomycin	Not tested	4 µg/mL (Susceptible)	≤0.5 µg/mL (Susceptible)	4 µg/mL (Susceptible)	8 µg/mL (Resistant)
Linezolid	Not tested	≤2 µg/mL (Susceptible)	≤2 µg/mL (Susceptible)	≤1 µg/mL (Susceptible)	≤1 µg/mL (Susceptible)
Vancomycin	4 µg/mL (Susceptible)	>16 µg/mL (Resistant)	>16 µg/mL (Resistant)	>16 µg/mL (Resistant)	>16 µg/mL (Resistant)
Gentamicin (High-level synergy)	≤500 µg/mL (Susceptible)	≤500 µg/mL (Susceptible)	Not tested	Not tested	Not tested
Streptomycin (High-level synergy)	≤1000 µg/mL (Susceptible)	≤1000 µg/mL (Susceptible)	Not tested	Not tested	Not tested
Tetracycline	Not tested	Not tested	Not tested	≤4 µg/mL (Susceptible)	>8 µg/mL (Resistant)
Eravacycline	Not tested	Not tested	Not tested	0.008 µg/mL (Susceptible)	0.008 µg/mL (Susceptible)

**Table 4:** MICs are reported in µg/mL with categorical interpretations based on CLSI breakpoints applicable at the time of testing broth microdilution. “Not tested” indicates antimicrobial susceptibility testing was not performed for that agent on the specified isolate.

Antimicrobial Agent	4 Days Post–Ex-Laparotomy: Aspirated Abdominal Fluid	10 Days Post–Ex-Laparotomy: Aspirated Abscess Fluid	10 Months Post–Initial Colectomy: Aspirated Abdominal Fluid	2.5 Years Post–Initial Colectomy: Aspirated Abdominal Fluid	2.5 Years + 10 Days Post–Initial Colectomy (Repeat): Aspirated Abscess Fluid
Ampicillin	>8 µg/mL (Resistant)	Not tested	>8 µg/mL (Resistant)	>8 µg/mL (Resistant)	Not tested
Daptomycin	2 µg/mL (Susceptible)	8 µg/mL (Resistant)	2 µg/mL (Susceptible)	2 µg/mL (Susceptible)	8 µg/mL (Resistant)
Linezolid	2 µg/mL (Susceptible)	Not tested	2 µg/mL (Susceptible)	2 µg/mL (Susceptible)	Not tested
Vancomycin	>16 µg/mL (Resistant)	Not tested	>16 µg/mL (Resistant)	>16 µg/mL (Resistant)	Not tested
Gentamicin	Not tested	Not tested	Not tested	Not tested	Not tested
Ciprofloxacin	Not tested	Not tested	Not tested	Not tested	Not tested
Meropenem	Not tested	Not tested	Not tested	Not tested	Not tested
Piperacillin–Tazobactam	Not tested	Not tested	Not tested	Not tested	Not tested

## Data Availability

No new data were generated for this study. All relevant clinical information is presented within the article. Additional details may be made available from the corresponding author upon reasonable request, subject to privacy restrictions related to patient confidentiality.
